# Why is motilin active in some studies with mice, rats, and guinea pigs, but not in others? Implications for functional variability among rodents

**DOI:** 10.1002/prp2.900

**Published:** 2022-02-21

**Authors:** Gareth J. Sanger

**Affiliations:** ^1^ Blizard Institute and the National Centre for Bowel Research Barts and The London School of Medicine and Dentistry Queen Mary University of London London United Kingdom

**Keywords:** animal variation, experimental reproducibility, motilin, pseudogene, rodent

## Abstract

The gastrointestinal (GI) hormone motilin helps control human stomach movements during hunger and promotes hunger. Although widely present among mammals, it is generally accepted that in rodents the genes for motilin and/or its receptor have undergone pseudonymization, so exogenous motilin cannot function. However, several publications describe functions of low concentrations of motilin, usually within the GI tract and CNS of mice, rats, and guinea pigs. These animals were from institute‐held stocks, simply described with stock names (e.g., “Sprague–Dawley”) or were inbred strains. It is speculated that variation in source/type of animal introduces genetic variations to promote motilin‐sensitive pathways. Perhaps, in some populations, motilin receptors exist, or a different functionally‐active receptor has a good affinity for motilin (indicating evolutionary pressures to retain motilin functions). The ghrelin receptor has the closest sequence homology, yet in non‐rodents the receptors have a poor affinity for each other's cognate ligand. In rodents, ghrelin may substitute for certain GI functions of motilin, but no good evidence suggests rodent ghrelin receptors are highly responsive to motilin. It remains unknown if motilin has functional relationships with additional bioactive molecules formed from the ghrelin and motilin genes, or if a 5‐TM motilin receptor has influence in rodents (e.g., to dimerize with GPCRs and create different pharmacological profiles). Is the absence/presence of responses to motilin in rodents’ characteristic for systems undergoing gene pseudonymization? What are the consequences of rodent supplier‐dependent variations in motilin sensitivity (or other ligands for receptors undergoing pseudonymization) on gross physiological functions? These are important questions for understanding animal variation.

**Abbreviations**
5‐HT: 5‐hydroxytryptamine
CNS: central nervous system
GI: gastrointestinal
LH: luteinizing hormone
MMC: migrating motor complex
TM: transmembrane

## 1 INTRODUCTION TO MOTILIN

In humans, the hormone motilin is found mostly within endocrine cells of the mucosa of the duodenum and jejunum, and to a lesser extent the gastric antrum.^1^, ^2^ Motilin is released from these cells during hunger to induce phase III activity of the gastric migrating motor complex, a wave of high amplitude and propulsive contractions which occurs during fasting every 90–120 min and moves from the stomach and into the small intestine. Its purpose is thought to help clear the stomach of any undigested material, prevent bacterial overgrowth, and stimulate a sensation of hunger.^3^, ^4^, ^5^ Intravenous infusions of motilin to humans have also been shown to stimulate the motility of the gastric antrum,^6^, ^7^ increase gastric emptying of a solid meal,^8^ and induce postprandial nausea.^7^ Experiments with human isolated stomach showed that the gastric prokinetic activity of motilin occurs primarily because of an ability to act prejunctionally within the enteric nervous system to strongly facilitate cholinergic activity in a concentration‐dependent manner, with the higher concentrations also directly contracting the muscle; the large magnitude of the excitatory nerve‐muscle responses at the higher concentrations, perhaps in conjunction with vagal nerve activation, have been argued to help promote the ability of high doses of motilin and of motilin receptor agonists to cause nausea and vomiting.^9^

## 2 LOSS OF A FUNCTIONAL MOTILIN SYSTEM AMONG RODENTS

Motilin is found within the mammalian kingdom, with orthologues identified in birds, reptiles, amphibians, and fishes; the receptor for motilin, a seven‐transmembrane (TM), G‐protein‐coupled structure (first identified in human^10^), has a matching presence.^11^, ^12^, ^13^ However, examinations of genomic databases, including those assembled by Ensembl, found that among the mammals, the genes in rodents (mouse, rat, kangaroo rat, guinea pig, squirrel, a strain of pika) for the motilin receptor and often for motilin itself, have become pseudogenes (e.g., 80%–90% identity to the human motilin receptor, but with an in‐frame stop‐codon). This indicates that in rodents the functions of motilin have been lost^11^, ^12^, ^14^, ^15^ For the mouse and rat, this is thought to have occurred via mutations in the genes encoding the motilin receptor and motilin, and not by a disruptive chromosomal rearrangement that potentially could have removed both genes.^11^ Interestingly, among the amphibians, the reverse may be true. Certain frogs (e.g., the tropical clawed frog may have retained a motilin receptor but lost the presence of motilin; the authors suggest the possibility of a different endogenous agonist acting at the motilin receptor).^13^

Among rodents, the evolutionary pressures that led to the elimination of the presence and functions of motilin are unclear. However, it has been speculated that because rodents have also lost the ability to vomit (with marked and associated changes in the presence of other genetic markers and in neuronal, hormonal, and structural functions regulating upper gastrointestinal (GI) functions) all, or many of these events may have been somehow driven by an environmental pressure for water conservation in arid or semi‐arid regions.^12^ Early pseudonymization of the motilin receptor was followed by loss of the motilin peptide, complete in some rodent species but not in others. For example, although a potentially functional form of motilin was not identified in the guinea pig by He et al,^11^ a later search of Ensembl Genome databases by Kitazawa et al^16^ confirmed a proposed existence of guinea pig motilin (two different structures were first identified in the Ensembl Genome Database by Xu et al^17^ confirmed by qPCR and by Southern blot hybridization), which when synthesized were inactive when applied to guinea pig GI muscle strips but were effective stimulants when applied in similar experiments using rabbit duodenum (the evoked activity was reduced by the motilin receptor antagonist GM‐109 and by human motilin desensitization). Interestingly the later RT‐PCR using various primer sets failed to amplify the mRNA for one of the putative motilin structures.^16^ Nevertheless, the differences in data obtained by different investigators suggested that the gene for motilin in guinea pigs is undergoing pseudogenization but highly divergent alleles of the gene exist within the cDNA and genomic sequences of the guinea pig population.^18^ Another study into the North American kangaroo mouse and rat (members of the *Dipodomyinae* subfamily of rodents) identified potentially functional forms of motilin but since the motilin receptor pseudogene was formed well before the radiation of this subfamily, the retention of a potentially functional motilin was suggested to represent a lineage‐specific physiological adaptation to a new function.^18^

## 3 THE RODENT ANOMALIES

In contrast with the failure to identify a functional motilin gene within genomic databases of rats and mice (see above), or identify the presence of motilin,^19^, ^20^ there are several publications in which motilin is reported to be present within these animals (recent examples include^21^, ^22^, ^23^). Furthermore, although the application of motilin has been found to be without activity in several experiments with stomach and intestinal preparations from rats, mice, and guinea pigs (examples include^16^, ^24^, ^25^, ^26^, ^27^, ^28^), several other publications report an ability of motilin to exert functional activity in the stomach, brain and other tissues of rats, mice, and guinea pigs (briefly noted previously^11^, ^29^). Table 1 lists these studies and when provided by the authors, gives the sources of rodents and ligands used within each investigation. Examination of Table 1 reveals several features:

- Most (but not all) studies reporting functions of motilin involve the GI tract and the central nervous system (CNS)
- Some studies used cultured or dispersed primary cell populations, including all those studying GI muscle functions
- The studies date from the 1980s to 2019
- Responses to motilin have been reported when using both outbred and inbred animal suppliers
- In vitro, the effective concentrations of motilin are often in the nM range, similar to the low concentrations which activate the human and rabbit motilin receptors
- In some studies, the effective concentrations are in the μM range suggesting potential activation of a non‐motilin receptor

**TABLE 1:** Responses to motilin and macrolides (mostly erythromycin) in rodents

Species	Animal supplier	Ligand	Ligand supplier	Response	References
Gastrointestinal responses to motilin					
Guinea pig (Hartley; male)	Not stated (USA)	Synthetic porcine motilin^a^	Peninsula Laboratories	Motilin elicited concentration‐dependent contraction of primary gastric smooth muscle cells (l0^−l2^ to l0^−6^ M; ED_50_ l0^−9^ M)	[30]
Guinea pig	Japan	Motilin		Motilin induced contraction of longitudinal and circular muscle cells from small intestine, in concentration‐dependent manner with ED_50_’s of 0.3 nM and 0.05 nM, respectively	[31]
Rat (Sprague–Dawley, male)	Not stated (USA)	Porcine motilin	Sigma	Intra‐aortic (10^−4^, 10^−3^ nmoles/kg) but not oral motilin accelerated upper GI transit but not gastric emptying	[32]
Rat	Beijing, China			Motilin (10^−11^ to 10^−10^ mol) elicited contraction of isolated smooth muscle cells of stomach	[33]
Rat (Sprague–Dawley; male, female)	Not stated (China)	Porcine motilin	Sigma	20 μg/kg induced premature phase III contractions of antral origin	[34]
Guinea pig (Hartley; male)	Not stated (Japan; same laboratory as below)	Motilin	Peptide Institute, Osaka	Depolarization of myenteric neurons at 10 nM and above	[35]
Guinea pig (Hartley; male)	Not stated (As above; Japan)	Motilin	Peptide Institute, Osaka	Motilin acted presynaptically to inhibit myenteric nerve fast excitatory postsynaptic potentials	[36]
Rat (Sprague–Dawley; male, female)	Animal facility of the Fourth Military Medical University, China	Porcine motilin	Sigma	Antral cells isolated and cultured from neonatal rats; motilin (10^−7^–10^−5^ M) increased intracellular [Ca^2+^] concentration	[37]
Mouse (Type not specified; male, female)	Institute of Cancer Research mice (Samtako Bio Korea Co., Ltd., Osan, Korea)	Motilin	Tocris Bioscience	Whole‐cell patch‐clamp. Motilin 1–5 μM depolarized interstitial cells of Cajal in concentration‐dependent manner, inhibited by ghrelin receptor antagonist [D‐Lys] GHRP‐6.	[38]
Central Nervous System responses to motilin					
Rat (Sprague–Dawley; female)	Not stated (Arizona, USA)	Motilin	Not stated	Intracerebroventricular (0.2–2 μg) or intrathecal (1–2 μg) but not peripheral (intraperitoneal, subcutaneous) motilin caused dose‐related inhibition of micturition reflex, reversed by naloxone	[39]
Rat (Sprague–Dawley male)	Holtzmann, USA	Motilin porcine	Peninsula labs (Lot no 002469)	Stimulated growth hormone release from dispersed anterior pituitary cells (10^−9^ to 10^−5^ M). Only high IV doses (100 μg/kg) increased circulating growth hormone release in vivo and infusion into the fourth ventricle suppressed release	[40]
Rat (embryos)	Not stated (Gubnar, Japan)	Motilin	Peninsula labs	Increased neuronal firing of dissociated brainstem neurons; 1 nM solution applied iontophoretically	[41]
Rat	Not stated (Wisconsin, USA)			Intraperitoneal injection of motilin into fasted, but not fed, rats stimulated eating in dose‐dependent manner at 5 and 10 μg/kg	[42]
Rat (Sprague–Dawley; male)	Sasco Labs, Inc., Madison, USA	Synthetic porcine motilin	Peninsula labs	Injection of 1 μg motilin into the intracerebroventricular space increased food consumption at 2, 22, 24 h	[43]
Mouse (ddY, male)	Japan Slc Inc.	Porcine motilin	Motilin: Peptide Research Institute, Osaka, Japan; GM‐109: Chugai, Japan	Increase in food intake 1 h after ICV motilin (3 nmol/mouse), attenuated by GM‐109, a motilin receptor antagonist	[44]
Mouse (ddY, male)	Japan Slc Inc., Hamamatsu, Japan	Porcine motilin	Protein Research Foundation, Peptide Institute (Japan); Chugai, Japan	Motilin decreased anxiety (elevated plus maze) with inverted U‐shaped dose–response, antagonized by GM‐109, a motilin receptor antagonist	[45]
Rat (Wistar‐Imamichi; female)	Not stated (Japan)	Porcine Motilin	Peptide Institute, Osaka, Japan	Intravenous motilin (37 nmol/rat) suppressed luteinizing hormone (LH) release and increased food intake in ovariectomized rats. Also suppressed LH secretion when centrally administered	[46]
Rat	China			Motilin 10 nM depolarized Purkinje cells of cerebellum; mimicked by erythromycin	[47]
Mouse (BalB/C; male)	Laboratory Animal Center of the Fourth Military Medical University, China	Human motilin; Erythromycin	ADI, USA; Biobasic, Canada; Chugai, Japan	Motilin 10 nM depolarized interneurons in amygdala slices and facilitated GABAergic transmission; mimicked by 1 μM erythromycin and blocked by MA−2029, a motilin receptor antagonist. Erythromycin or motilin into the basolateral nucleus reduced anxiety‐like behavior	[48]
Mouse (C57/BL6J; male, female)	CLEA, Japan	Motilin	Sigma‐Aldrich	Motilin (0.1 μM) decreased discharge frequency of spontaneous action potentials in vestibular nuclear neurons and enhanced amplitudes of inhibitory postsynaptic currents	[49]
Rat	Qingdao, China			Neurons in dorsal vagal complex responsive to gastric distension, excited by microinjection of motilin, together with increased amplitude of gastric contractions after intracerebroventricular administration	[50]
Rat	Qingdao, China			Neurons in lateral hypothalamus responsive to gastric distension, excited or inhibited by microinjection of motilin, together with increased gastric antrum motility index	[51]
Rat (Wistar; male, female)	Qingdao Marine Drug Institution, China	Motilin	Supplied by Dr. Peeters, Leuven, Belgium	Neurons in CA3 region of hippocampus responsive to gastric distension, excited by microinjection of motilin, together with increased amplitude of gastric contractions after intracerebroventricular administration	[52]
Rat (Wistar; male)	Qingdao Marine Drug Institution, China	Rabbit Motilin	Motilin: Eurogenetics. Gent, Belgium; GM‐109: Chugai Pharmaceuticals	Neurons in basomedial amygdala nucleus responsive to gastric distension, inhibited or excited by microinjection of motilin, together with increased amplitude of gastric contractions after intracerebroventricular administration; GM‐109, a motilin receptor antagonist, had opposite activity	[53]
Rat (Wistar; male)	Qingdao Marine Drug Institution, China	Rabbit Motilin	Not stated	Micro‐pressure injection of 20 nM solution of motilin exited hippocampal neurons; responses blocked by application of GM‐109, a motilin receptor antagonist	[54]
Rat (Wistar; male)	Institute of Pharmaceutical Research of Qingdao, Qingdao, China	Rabbit Motilin	Supplied by Dr. Peeters, Leuven, Belgium	Neurons in arcuate nucleus responsive to gastric distension, activated by local administration of motilin in nM concentrations, together with increased frequency and amplitude of gastric contractions; abolished by GM‐109, a motilin receptor antagonist	[55]
Other responses					
Rat (Sprague–Dawley; male)	Not stated	Porcine Motilin	Peninsula labs	Motilin 30–300 nM/kg produced prolonged depressor response without affecting heart rate	[56]
Rat (Sprague–Dawley; male)	Charles River Laboratories, Canada	Motilin (human/porcine sequence)	Peptidec Technologies (Montreal, Canada)	In primary adipocytes, motilin 1 nM increased fatty acid and glucose uptake. In a cell line (murine preadipocyte from American Type Culture Collection), response to motilin mediated via ghrelin or motilin receptor	[57]
Gastrointestinal responses to erythromycin and other macrolides					
Guinea pig (male)	Morini (Monza, Italy).	Motilin and erythromycin	Motilin: Peninsula Laboratories; Erythromycin: Sigma	Stimulation of pepsinogen secretion from gastric chief cells by motilin 1–100 pM or erythromycin 1 pM−1 nM	[58]
Mice (C57 black, male)	Jackson Laboratories (USA)	Erythromycin	Sigma or Baker	Erythromycin accelerated gastric emptying (phenol‐red‐labeled saline with 20% dextrose)	[59]
Rat (Lew/SsNHsd Sprague–Dawley)	Harlan UK	ABT−229	Provided by Abbott Laboratories, UK.	ABT‐229 induced more propagated activity fronts in jejunum during morphine‐induced dysmotility	[60]
Central Nervous System responses to erythromycin and other macrolides					
Rat (Sprague–Dawley; male)	Qingdao Institute for Drug Control, China	Erythromycin	Not stated	Intracerebroventricular erythromycin (91.56 nmol, i.c.v.) stimulated gastric motility of diabetic rats, blocked partially by the motilin receptor antagonist GM‐109, a motilin receptor antagonist	[23]
Rat (Sprague–Dawley; male, female)	Institute for Family Planning, Shanghai, China	Erythromycin	Sigma‐Aldrich	Erythromycin 100 nM −10 µM inhibited the frequency of glycinergic spontaneous inhibitory postsynaptic currents of gastric vagal motorneurons and inhibited amplitude at 10 µM. Responses prevented by GM−109	[61]
Other responses to erythromycin and other macrolides					
Rat (Wistar)	Animal Room of Lanzhou Medical College China.	Erythromycin	Sigma	Erythromycin (5 × 10^−5^–1.55 × 10^−3^ M) increased contractile frequency of uterine smooth muscle strips from non‐pregnant rats, and at 1.55 × 10^–3^ mol/L, increased muscle tension in uterine muscle from non‐pregnant rats	[62]

Note

Within each section the studies have been listed by the date of publication, beginning with the oldest first. The exception is the collection of studies on the functions of motilin within the CNS from Qingdao, China (references 100–105), which are grouped together in order of date of publication (also reference 111 which refers to erythromycin).

^a^ Porcine motilin identical to human motilin [63].

The motilin receptor may also be activated by the anti‐biotic drugs erythromycin and azithromycin,^64^ and by other drugs with similar, macrolide structures.^65^ These drugs stimulate upper GI motility and are used “off label” to increase gastric emptying for therapeutic ultility.^3^, ^63^ Accordingly, Table 1 also lists a smaller number of studies in which functions of the anti‐biotic drug erythromycin or related structures have been identified within rodents. However, these drugs may possess uncertain additional pharmacology. There are, for example, several reports of an inhibitory activity of high concentrations or doses of erythromycin in GI and other smooth muscle preparations, not mediated by activation of the motilin receptor.^66^, ^67^, ^68^, ^69^, ^70^ In addition, the compound EM574, a derivative of erythromycin, can activate the ghrelin receptor (IC_50_ of 10 mM^71^). More recently, it has been suggested that the anti‐inflammatory actions of erythromycin in chondrocytes may be mediated directly or indirectly via the ghrelin receptor.^72^ This complexity of activity among the macrolides makes it difficult to further consider the mechanisms by which these structures exert activity in rodents. For this reason, only the functions of motilin itself are now discussed.

How can motilin have activity in some studies with rodents but not in others? In attempting to answer this question it is difficult to avoid the possibility that the variation is somehow related to the source and/ or type of animal used, which introduces important variations in genetic coding and potentially, in receptors and pathways by which motilin can exert function.

## 4 SOURCE‐ AND STRAIN‐DEPENDENT DIFFERENCES AMONG RODENTS

In the experiments in which a function of motilin was identified, the sources of animals were not always provided (Table 1). Where this information was given, analysis showed that animals were from institute‐held stocks (e.g., Institute of Cancer Research mice, Qingdao Marine Drug Institution), or were described simply by stock names such as “Hartley” (guinea pig), “Sprague–Dawley,” and “Wistar” (rats), or sometimes, the authors used inbred strains of mice (eg C57/BL6J). An advantage of inbred strains of mice is that they are thought to minimize genetic variability between individual animals (but see Tuttle et al,^73^ who found evidence of high genetic variability), although these animals may be relatively small and subject to selection pressures which favor adaptation to captivity (e.g., social behaviors^74^). The use of stock names does not signify a known genomic identity, which may differ between the same type of animal from different breeders. Such animals (also highly inbred) can be subject to founder effects and genetic drift and may show substantial genetic divergence from other colonies.^75^

Many genetic differences exist between different strains of mice. An investigation into 17 different mouse genomes (including classic laboratory strains and the progenitors of strains linked to more than 5000 different types of knockout mice), identified 56.7 million unique single nucleotide polymorphisms, 8.8 million unique indels (insertion or deletion of nucleotide bases), and 0.28 million structural variants.^76^ These differences may be associated with differences in functions. Examples of GI functions in which strain‐dependent differences are reported include differences in expression of L‐Tryptophan hydroxylase 2 (tph2) gene polymorphism within the intestine, in the numbers of close contacts between different phenotypes of enteric neurons and in the sensitivities of muscle contractions to 5‐hydroxytryptamine (5‐HT).^77^ In addition, clear differences have been reported in the propensity of different strains to defecate or release colonic 5‐HT,^78^ in the sensitivities of different strains of mice to pica behavior induced by cisplatin,^79^ and in the thickness of the stomach wall, frequency of duodenal contractions and rate of defecation of an ingested marker.^80^

Might the variations in genetic structures of rodents from different sources explain why some studies find no ability of motilin to exert function, whereas others report a function? Without a rigorous examination of those animals in which a response was found, this question is impossible to answer. Nevertheless, certain speculations seem reasonable.

## 5 POTENTIAL MOLECULAR DIFFERENCES BETWEEN ANIMALS FROM DIFFERENT SOURCES

### 5.1 Motilin receptor

As yet, there is no evidence for a functional motilin receptor among rodents. In each species examined by He et al^11^, ^18^—rat, mouse, guinea pig (confirmed by Sanger et al^12^ in similar experiments), and animals that are not typical laboratory species (squirrel, pika, kangaroo rat, and mouse)—a potentially functional motilin receptor was not identified. In one other study, motilin receptors were identified within the myenteric plexus of guinea pig ileum by immunohistochemistry, but the receptor mRNA was not found by qPCR^22^; these conflicting data were suggested by the authors to have occurred because the receptor was structurally distinct from the human receptor on which the primers were designed.

In no other experiment in which a response to motilin was detected, have attempts been made to isolate the motilin receptor by qPCR or other techniques. Accordingly, it remains a possibility that in some populations of rodents a functional motilin receptor exists. However, an alternate possibility is that a different receptor has appeared with good affinity for motilin, capable of eliciting a functional response. The current absence of an identified rodent motilin receptor favors this second possibility which if correct, indicates the existence of a past or present evolutionary pressure to retain the functions of motilin and generate a motilin‐sensitive receptor; if endogenous motilin is no longer present then an ability to respond to exogenously applied motilin would represent a vestigial sensitivity. Notably, in the study by He et al^18^ into the North American kangaroo mouse and rat, the retention of a potentially functional motilin was suggested to represent a lineage‐specific physiological adaptation to a new function.

Could a non‐motilin receptor, sensitive to motilin, exist within the cDNA and genomic sequences of the rodent population? The receptor with the closest sequence homology is the ghrelin receptor.

### 5.2 Ghrelin receptor

The motilin and ghrelin receptors belong to the same sub‐family of 7‐TM GPCRs, sharing significant amino acid identities in different species (e.g., the human motilin and ghrelin receptors and the receptors in the insectivore *Suncus murinus* each share, respectively, 52% and 42% overall amino acid identity and 86% and 62% in the seven‐transmembrane region^81^, ^82^, ^83^). Both hormones are released from endocrine cells of the upper GI tract at different times during fasting and both stimulate gastric motility and have roles in the feeding cycle in humans and other mammalian species; unlike motilin, ghrelin is also found outside the GI tract where it can exert significant additional non‐GI functions.^84^, ^85^ Might the ghrelin receptor substitute for the absence of a functional motilin system? This seems to be a possibility in terms of the control of gastric functions, but good evidence to suggest that the rodent ghrelin receptor is highly responsive to exogenous (or endogenous) motilin is lacking.

In rodents, it has been suggested that the absence of a functional motilin system is compensated for by the actions of ghrelin.^29^, ^86^ This may be illustrated by the species‐dependent roles of motilin and ghrelin in the mechanisms of the migrating motor complex (MMC). In humans, the release and subsequent actions of motilin during fasting mediate the propulsive phase III contractile activity of the gastric MMC, also associated with hunger (see Introduction). Although ghrelin is released during fasting in humans, this is not in association with phase III MMC activity, its purpose being to increase appetite.^4^ In the insectivore *Suncus murinus* (house musk shrew), also possessing both motilin and ghrelin functional systems, the ability of motilin to induce phase III of the gastric MMC may involve the release of ghrelin.^87^, ^88^ In rats and mice, however, in which gastric MMCs are less well defined and more frequent, it is the release of ghrelin which evokes the phase‐III‐like contractions.^89^, ^90^, ^91^ Notably, ghrelin can directly stimulate gastric enteric nerve functions in rat and mouse, but not in human.^27^, ^92^, ^93^ Curiously, in rats with a mutant, non‐functional ghrelin receptor, spontaneous gastric phase III‐like contractions were still observed, suggesting the development of a different compensatory mechanism to maintain these contractions.^94^

In species possessing both motilin and ghrelin, the receptors have a poor affinity for each other's cognate ligand (e.g., the human and rabbit receptors^29^, ^95^). In mice, it has been suggested that the ghrelin receptor is responsive to motilin at high concentrations. Thus, using a whole‐cell patch‐clamp configuration, motilin 1–5 µM depolarized the pacemaker potentials of the interstitial cells of Cajal within the small intestine, in a concentration‐dependent manner; this activity was inhibited by the ghrelin receptor antagonist [D‐Lys] GHRP‐6.^38^ However, it is important to note that in most other in vitro studies in which motilin has been shown to exert activity in rodents, the efficacy is reported at nM concentrations (Table 1), similar to the concentrations which activate the human and rabbit motilin receptors.^29^, ^95^

### 5.3 Other possibilities

Additional bioactive molecules are formed from the ghrelin gene and possibly the motilin gene, potentially able to interact with receptors and in rodents, potentially interacting with motilin. This possibility has not been investigated. The first is des‐acyl ghrelin, formed from pre‐pro ghrelin and by de‐acylation of circulating ghrelin to activate a putative receptor (not yet molecularly identified) which appears to be poorly responsive to ghrelin and has been called the unacylated ghrelin or UAG receptor; evidence also exists for a further putative receptor at which ghrelin and des‐acyl ghrelin have similar potency.^96^, ^97^

Second, the predicted endoproteinase cleavage sites within the ghrelin and motilin genes are thought to generate additional peptides.^98^ For the ghrelin gene, this can generate obestatin, a peptide with biological activity (but with little or no ability to modulate rat GI motility^99^) and as yet, without a confirmed receptor.^100^ Other ghrelin gene splice variants include a C‐terminus truncated form of ghrelin, present in mice and humans.^101^ For the prepromotilin gene of motilin an additional cleavage site may generate a motilin‐associated peptide at the carboxy‐terminal, thought to play a role in protein degradation and posttranslational processing of motilin.^102^ Furthermore, a preliminary report suggested that a 17‐residue peptide (H‐Leu‐Thr‐Ala‐Pro‐Leu‐Glu‐Ile‐Gly‐Met‐Arg‐Met‐Asn‐SerArg‐Gln‐Leu‐Glu‐OH), similar in length to obestatin, may be generated by cleavage of the motilin gene, this peptide weakly mimicking the ability of motilin to increase cholinergically mediated contractions in rabbit isolated gastric antrum.^103^

Finally, a 5‐TM motilin receptor has been identified,^10^ with no known function. Similarly, a 5‐TM ghrelin receptor, without sensitivity to ghrelin, is able to dimerize with the ghrelin receptor, changing its function and ability to form oligomeric complexes with the dopamine D_1_ receptor, to create different pharmacological profiles.^104^

## 6 CONCLUSIONS AND QUESTIONS

The absence of genes generating motilin and/ or its receptor, and the absence of a functional response to motilin in laboratory rodents has become the accepted status for motilin. Nevertheless, confusion remains over numerous reports, which demonstrate an ability of low concentrations of motilin to exert functional activity in some laboratory rodents, particularly within the GI tract and the CNS. There is no accepted explanation for this anomaly, but the very existence of such differences raises concerns, particularly in terms of the need to understand animal research reproducibility.^105^

It is difficult to refute the suggestion that the variation in response to motilin is dependent on the source of rat, mouse, or guinea pig used. This includes outbred animals and genetically stable in‐bred strains of mice. The cause of the variation remains unknown, but it can be speculated that molecular differences in the receptors for motilin, ghrelin and perhaps for associated peptides might be involved. If correct, several questions need to be asked.

1. Is the variation in response to motilin characteristic for functions that are undergoing gene pseudonymization?
  In different species of rodent, He et al^11^, ^18^ described the complete loss of functional genes for motilin and its receptor, but in others, a functional motilin gene remained whilst the receptor was non‐functional (the opposite may be true in certain amphibians^13^). This variation was argued to have been brought about by early pseudonymization of the motilin receptor followed by progressive pseudonymization of the motilin gene during the evolution of the *Rodentia* order.
  - In some laboratory rodent strains, could differences in genomes between animals from different outbred suppliers include the retention of a functional motilin receptor? Studies are needed to look for the motilin receptor in animals which respond to motilin.
  - Since pseudonymization of the motilin receptor gene occurred before the loss of the motilin gene is it possible that a different receptor has evolved to respond to motilin? The effects of motilin on the functions of receptors closely related to motilin should be investigated. This includes the rodent ghrelin receptor (e.g., has the affinity of the ghrelin receptor for motilin increased, such that nM concentrations of motilin are now able to activate the receptor?), and when identified, the putative receptors activated by other bioactive peptides generated from the ghrelin and possibly the motilin gene.
  - What are the consequences of gene pseudonymization for other receptor systems? Wang et al^106^ identified a variety of human pseudogenes, including those involved with chemoreception and immunity, but the physiological and pharmacological consequences of their progressive pseudonymization during mammalian evolution remain to be examined.
2. What are the consequences of rodent supplier‐dependent variations in sensitivity to motilin (or ligands for other genes undergoing pseudonymization) on activities of non‐motilin ligands involved in the same physiological functions as motilin (in non‐rodents or rodents exhibiting functional sensitivity to motilin)? An example of the actions of one endogenous ligand compensating for the loss of another is provided by Adkins et al,^107^ who found 10 times the normal level of insulin in the circulation of guinea pigs, speculating that since insulin possesses growth‐promoting activity it may be compensating for an absence of the functions of growth hormone, perhaps via the insulin‐like growth factor I receptor. With regard to motilin, the GI sites of action and functions of ghrelin in rodents may have upregulated to compensate for the absence of motilin (see earlier discussion). In addition, 5‐HT plays a role in MMC activity of mammals, including rodents.^108^ In humans 5‐HT_3_ receptor antagonists prolong the interval between successive MMCs but have no effects on gastric emptying of food.^108^, ^109^ Studies in dogs show that motilin and 5‐HT interact in a positive manner to facilitate the release of both mediators and their abilities to initiate the MMC cycle (involving 5‐HT_3_ receptors for MMCs originating in the stomach and 5‐HT_4_ receptors for MMCs originating in stomach and duodenum) and stimulate motilin release to sustain phase III activity.^110^ By contrast, in rodents the 5‐HT_3_ receptor is not involved in regulating MMC activity^111^ but 5‐HT_3_ receptor antagonists increase gastric emptying in rats and guinea pigs.^112^ Thus, if different populations of rodents have lost or still retain an ability to respond to motilin, it seems reasonable to suggest that similar variability will be found among the actions of other endogenous ligands involved with the same physiological functions as motilin.
3. Are motilin‐responsive rodents useful “knock‐in” laboratory animals for studying the functions of motilin? This would avoid having to rely on other non‐rodent species or rodents in which the human motilin receptor gene has been knocked‐in [29].

In summary, the existence of responses to motilin in rodents for which there is no demonstrated motilin receptor raises important questions relating to rodent research reproducibility, motilin research and potentially, in other areas of pharmacology where similar inconsistencies occur, perhaps where there is evidence of gene pseudonymization.

### 6.1 Nomenclature

Key protein targets and ligands in this article are hyperlinked to corresponding entries in the IUPHAR/BPS Guide to PHARMACOLOGY http://www.guidetopharmacology.org and permanently archived in the Concise Guide to PHARMACOLOGY 2021/22.^113^

## ACKNOWLEDGEMENT

The author would like to thank everyone who generated the data discussed in this manuscript and in particular, his laboratory colleagues and friends in other institutions who have participated in trying to understand why the pharmacology and physiology of mice and other rodents are not always the same as humans.

## CONFLICT OF INTEREST

The author has no conflict of interest with respect to this study.

## DISCLOSURE

None.

## AUTHOR CONTRIBUTION

GJS wrote this manuscript.

## Data Availability

This article contains no new data (other than a summation of the work of others).
